# A landscape for drug-target interactions based on network analysis

**DOI:** 10.1371/journal.pone.0247018

**Published:** 2021-03-17

**Authors:** Edgardo Galan-Vasquez, Ernesto Perez-Rueda

**Affiliations:** 1 Departamento de Ingeniería de Sistemas Computacionales y Automatización, Instituto de Investigación en Matemáticas Aplicadas y en Sistemas, Universidad Nacional Autónoma de México, Ciudad Universitaria, México City, México; 2 Instituto de Investigaciones en Matemáticas Aplicadas y en Sistemas, Universidad Nacional Autónoma de México, Unidad Académica Yucatán, Mérida, Yucatán, México; Utrecht University, NETHERLANDS

## Abstract

In this work, we performed an analysis of the networks of interactions between drugs and their targets to assess how connected the compounds are. For our purpose, the interactions were downloaded from the DrugBank database, and we considered all drugs approved by the FDA. Based on topological analysis of this interaction network, we obtained information on degree, clustering coefficient, connected components, and centrality of these interactions. We identified that this drug-target interaction network cannot be divided into two disjoint and independent sets, *i*.*e*., it is not bipartite. In addition, the connectivity or associations between every pair of nodes identified that the drug-target network is constituted of 165 connected components, where one giant component contains 4376 interactions that represent 89.99% of all the elements. In this regard, the histamine H1 receptor, which belongs to the family of rhodopsin-like G-protein-coupled receptors and is activated by the biogenic amine histamine, was found to be the most important node in the centrality of input-degrees. In the case of centrality of output-degrees, fostamatinib was found to be the most important node, as this drug interacts with 300 different targets, including arachidonate 5-lipoxygenase or ALOX5, expressed on cells primarily involved in regulation of immune responses. The top 10 hubs interacted with 33% of the target genes. Fostamatinib stands out because it is used for the treatment of chronic immune thrombocytopenia in adults. Finally, 187 highly connected sets of nodes, structured in communities, were also identified. Indeed, the largest communities have more than 400 elements and are related to metabolic diseases, psychiatric disorders and cancer. Our results demonstrate the possibilities to explore these compounds and their targets to improve drug repositioning and contend against emergent diseases.

## Introduction

The increase of *de novo* drug discovery has been significant in recent decades, although the pace of advances in genomics and pharmacogenomics has decreased as a consequence of a lack of investment in pharmaceutical research and development [1,2]. In addition, the long process to release a new drug to the market, once it is approved by the FDA, is an expensive process for the pharmaceutical industry [3,4]. In light of these challenges, exhaustive analysis based on existing drugs has emerged as a strategy to improve the positioning of drugs for which new clinical indications have been identified can decrease development risks and costs, as well as shorten the time between drug discovery and availability on the market [5,6].

To date, the collection of drugs and their targets have been deposited in diverse databases, and it is known that most drug targets are cellular proteins, such as enzymes, G-protein-coupled receptors (GPCRs), ion channels, transporters, and nuclear hormone receptors. The main goal of these collections is to treat or diagnose a disease through selective interaction with chemical compounds [7,8]. In general, on the basis of ligand-binding studies, these drug targets can be grouped into ~130 protein families; whereas ~3,000 targets for small-molecule drugs have been predicted for the human genome [9].

Conventionally, the discovery of drugs has followed the well-accepted paradigm of one drug—one target—one disease, which searches to identify the most specific drugs to act against a specific target for an individual disease [10]. However, diseases are caused by complex biological processes, which can be resistant to the activity of any single drug. This approach is called “polypharmacy” and has modified the conventional paradigm to multi-drugs—multi-targets—multi-diseases [11]. There is debate on the use of each of these approaches, as the “one drug—one target” paradigm can lead to highly potent and specific (single-target) treatments that may be better tolerated due to the absence of off-target side effects [12]. In contrast, the multi-drug approach is based on an understanding that, due to compensatory mechanisms and redundant functions, biological systems might develop resistance to the disturbances caused by a single drug [13].

This new paradigm has resulted on one hand in a significant decrease in the rate that new drug candidates are being translated and, on the other hand, in an increase in studies focused on new targets for existing drugs. These approaches include the drug side-effect similarity, disease networks, chemical structure information, protein-protein sequence similarity, drug-target interactions, and integrative networks propagation methods, among others [14–17].

Recently, diverse computational methods have been proposed to analyze datasets of interactions. In particular, the interactions of drugs and targets can be conceptualized in the form of a network, that conventionally has been described with a bipartite network structure, i.e., is a graph where the vertices can be divided into two disjoint sets such that all edges connect a vertex in one set to a vertex in another set [18–20]. In complex networks such as this, different topological measures allow describing underlying properties of biological relationships, such as, node degree that identifies the most connected nodes; node centrality that identifies relevant nodes in the networks; or communities that are subsets of densely connected nodes that may be related to similar biological processes. In this context, diverse metrics have been proposed to identify non-overlapping [21–23] and overlapping communities [24–26], where each node belongs to a unique cluster or each node is allowed to belong to more than one community at the same time, respectively.

In this work, we evaluated the global interactions between drugs and their targets to determine how connected are the compounds. This study is based on the assumption that similar drug molecules are likely to interact with similar targets. To this end, the interactions downloaded from the DrugBank database, considering drugs approved by the FDA, were obtained and analyzed exhaustively. From this information, diverse findings are reported, such as the network is not bipartite, *i*.*e*., it cannot be divided into two disjoint and independent sets. In addition, the connectivity or associations between every pair of nodes shows that the drug-target network is constituted by 165 connected components, where one giant component contains 4376 interactions that represent 89.99% of all the elements. The histamine H1 receptor, which belongs to the family of rhodopsin-like GPCRs and is activated by the biogenic amine histamine, was found as the most important node in the centrality of input-degrees. In the case of the centrality of output-degrees, fostamatinib was found to be the most important node, with interactions with 300 different targets, including arachidonate 5-lipoxygenase. The top 10 hubs interact with 33% of the target genes. From these, fostamatinib stands out since it is used for the treatment of chronic immune thrombocytopenia in adults. Finally, highly connected sets of nodes can be structured in communities, and the largest communities have more than 400 elements; these are related to metabolic diseases, psychiatric disorders and cancer.

## Materials and methods

### Dataset of drugs and targets

In order to analyze the interactions of drugs and their targets, we searched for data from the DrugBank database (https://www.drugbank.ca/; version 5.1.4, released 2019-07-02), which includes 2634 drugs [27]. The database comprises DrugCard entries with more than 200 data fields, with half of the information devoted to drug/chemical data and the other half devoted to drug target or protein data. From these entries, we only considered targets because they are defined as macromolecules or small molecules to which a given drug interacts with, resulting in an alteration of the normal function of the bound molecule and desirable therapeutic effects or unwanted adverse effects. We excluded carriers, transporters and enzymes because they are not altered in their functions as occurs in targets. For instance, a carrier binds to a drug and modifies its pharmacokinetics or may facilitate transport in the bloodstream or across cell membranes. A transporter refers to a kind of endogenous, membrane-bound, protein-based structure that physically moves drugs across cell membranes into and out of cells; and an enzyme facilitates the occurrence of a metabolic reaction by interacting with and transforming a drug or chemical to one or more specific metabolites. Therefore, we considered a dataset of 2689 different targets interacting with 2186 FDA-approved biotech drugs in the United States.

### Topological analysis

We applied the node connectivity to examine the network topological property difference of drugs and targets. In this regard, the drug-target interactions were defined as a directed network described by the equation *G =* (*V*, *E*), where nodes (*V*) represent drugs and targets and the edges (*E*) represent their interactions. Then, the network was characterized by topological metrics, such as node degree, clustering coefficient, centrality, hubs, and communities [28].

In brief, the degree of a node (*K*) is defined as the number of interactions that it has with other nodes. In directed networks, input degree (*K*_in_) and output degree (*K*_out_) are defined as the number of arrows that enter and leave a node, respectively; these values correspond to the number of drugs that affect a certain target and the number of targets that a drug can affect. It is known that many biological networks present a degree distribution that follows the approximate form of a power-law distribution, *P*(*k*) *= Ax*^−γ^, where *A* is a constant that warrants that the *P*(*k*) values are less than one and the degree exponent γ is often between 2 and 3 [29]. This distribution is characterized by the fact that most nodes have few connections and a small set of nodes are highly connected; these high degree nodes, termed hubs, are topologically and functionally important to the network structure.

The clustering coefficient *C*_*i*_ of the node *v*_*i*_ was calculated as follows: *C*_*i*_ = 2*Ei*/(*k*_*i*_)(*k*_*i*_-1), where *E*_*i*_ is the number of edges between the neighbors of *v*_*i*_ and indicates the probability that two nodes with a common neighbor in a graph are interconnected, *i*.*e*., it quantifies the extent to which the local neighborhood of a node is as a member of a group of nodes. In this context, the clustering coefficient takes values between 0 and 1, where 1 means that we found nodes whose neighbors are connected among them forming complete graphs [28]. The shortest path length is the minimal number of edges needed to reach a node from the other node through a path along the edges of the network [28].

Centrality is a function, *C*, which assigns every *v* ∈ *V* of a given graph *G* the value C(v) ∈ *R*. As we are interested in the ranking of the node of the given graph *G*, we chose the convention that node *u* is more important than another node, *v*, if *C*(*u*) > *C*(*v*). There are many measures of centrality, including degree, closeness, betweenness, and eigenvector centrality.

The simplest centrality is the *degree* centrality, which gives for every vertex *v* a measure of the relative connectivity of a vertex in the network [28]. It is calculated as the degree of the vertex over *n-1*, this is the maximum possible degree in a network with *n* vertices [28].

*Closeness* centrality of a vertex *v* is defined as the reciprocal of the sum of the length of the shortest paths between the vertex *v* and all other vertices *u* in the graph, and it is calculated as:

$$C_{Clo}(v)=\frac{n-1}{\sum_{V-1}^{n-1}d(u,v)}$$

where *d(u*,*v)* is the shortest-path distance between *v* and *u*, and *n* is the number of vertices in the network. The largest value indicates the node that minimizes the sum of all distances to all other nodes [28].

The *betweenness* centrality of a vertex *v* is the sum of the fraction of all-pairs shortest paths that pass-through *v*, it is calculated as

$$C_{Bet}(v)=\sum_{st,\in V}^{\cdot }\frac{\sigma (s,t|v)}{\sigma (s,t)}$$

where *σ*(*s*,*t|v*) denote the number of shortest paths between *s* and *t* that use *v* as an interior vertex. The highest values correspond to nodes that best measure the ability of a vertex to monitor communication between other vertices, a value of zero is assigned to nodes that do not participate as interior vertices in any shortest path communication between other vertices [28].

*Eigenvector* centrality designates the significance of a vertex proportionally to the importance of their neighbors, then a vertex is significant because it is connected to many vertices or because it is connected to vertices with large eigenvector centralities. The value of this centrality in each vertex *v* is obtained by using the adjacency matrix *B*, their largest eigenvalue *λ*_*n*_ and an initial vector *x*_*v*_(0), we get the value in *v* by the iteration of the function

$$x_{v}(t+1)=\frac{1}{\lambda_{n}}\sum_{u}^{\cdot }.\;Bx_{v}(t)$$

The sum is over all the vertices *u* in the network. A connected network ensures that we can obtain a fixed value *x*_*v*_ after a finite number of iterations. A usual *x*(0). in computational algorithms is the eigenvector associated with *λ*_*n*_. This centrality gives values for every vertex and the vertices that are reachable in less steps receive a low value. On the other hand, high centrality value for central vertices that require more steps [28].

### Connectivity

The connectivity in a network refers to the associations between each pair of nodes. These connections can be via a direct link or indirect through a series of intermediate connections. In this context, the connected component is a set of nodes that are linked to each other by paths, and this gives us information about how connected the elements in a network and their module structure are [28].

### Community identification

Given a network *G*, a community *C* is defined as a set of distinct nodes: *C* = {*v*_1_,*v*_2_,…,*v*_*n*_}, where *n* is the node in the network. Many metrics tend to be described to identify communities [30–32], we used the multilevel algorithm described by [23], which assigns a different community to each node of the network, and then a node is moved to the community of one of its neighbors with which it achieves the highest positive contribution to modularity. The above step is repeated for all nodes until no further improvement can be achieved. Then, each community is considered a single node on its own and the second step is repeated until there is only a single node left or when the modularity cannot be increased in a single step. To this end, we use a random decomposition of the nodes and a resolution of one. This parameter changes the size of the identified communities [33].

### Functional annotation analysis

To identify the disease, and disease class, and metabolic pathways enriched in each set of nodes as hubs and communities, we used the Database for Annotation, Visualization and Integrated Discovery (DAVID; http://david.abcc.ncifcrf.gov/), which is a gene functional classification system that integrates a set of functional annotation tools [34]. We refer as enrichment analysis to the statistical method which identifies groups of targets or drugs that are over-represented in a large set of ranked elements, and may be related to diseases.

### Algorithms and implementation

**Fig 1:**
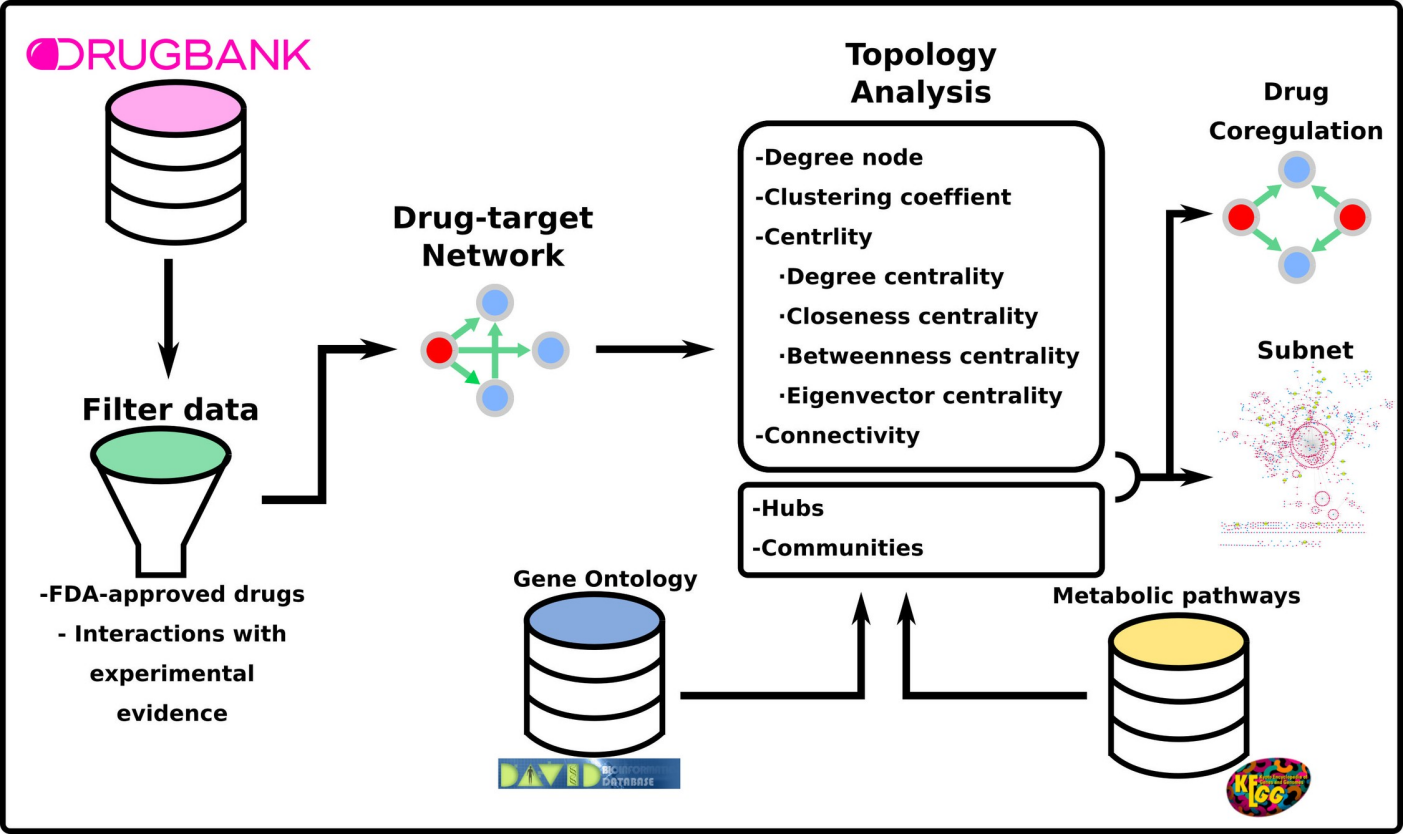
Schematic workflow of the drug-target network analysis. See text for details.

Algorithms of degree, degree distributions, clustering coefficient, centralities and communities were implemented in Python 3.6 (https://www.python.org/) and coregulations and dominate sets were implemented in Octave (https://www.gnu.org/software/octave/), and are available at S1 File. In Fig 1, we show the schematic workflow for the analysis.

## Results and discussion

### General features of the network of drug-target interactions

In order to evaluate the associations between drugs and their targets, the information obtained from the DrugBank database was organized into a target interaction network. To this end, we considered the interactions approved by the FDA and commercialized in the U.S. From this approach, a total of 9286 interactions between 2186 drugs and 2689 targets were analyzed (S1 Table). It is interesting that adenosine, cystine, copper, dabigatran etexilate, digoxin, glutathione, iron, lactose, nitric oxide (NO), methotrexate, prothrombin, somatostatin, and tyrosine compounds were identified as acting as both drugs and targets. For instance, NO that acts as a neuromodulator, regulating the hypothalamic release of neuropeptides [35], was identified by one side, as a drug of three targets, Guanylate cyclase soluble subunit alpha-2 (with an Heme binding function), Metallothionein-1A (a zinc ion binding), and Indoleamine 2,3-dioxygenase 1 (Tryptophan 2,3-dioxygenase activity); and by other side, is target of albumin that results from its contribution to plasma colloid oncotic. Probably, albumin form can be used as a NO traffic protein [36–38].

This finding suggests that the network is not bipartite, *i*.*e*., it cannot be divided into two disjoint and independent sets. Therefore, based on the interaction network, topological analyses were achieved and are discussed in the following paragraphs, including their degree, clustering coefficient, connected components, and centrality.

### Network topological analysis: Degree, clustering coefficient, connected components, and centralities

**Fig 2:**
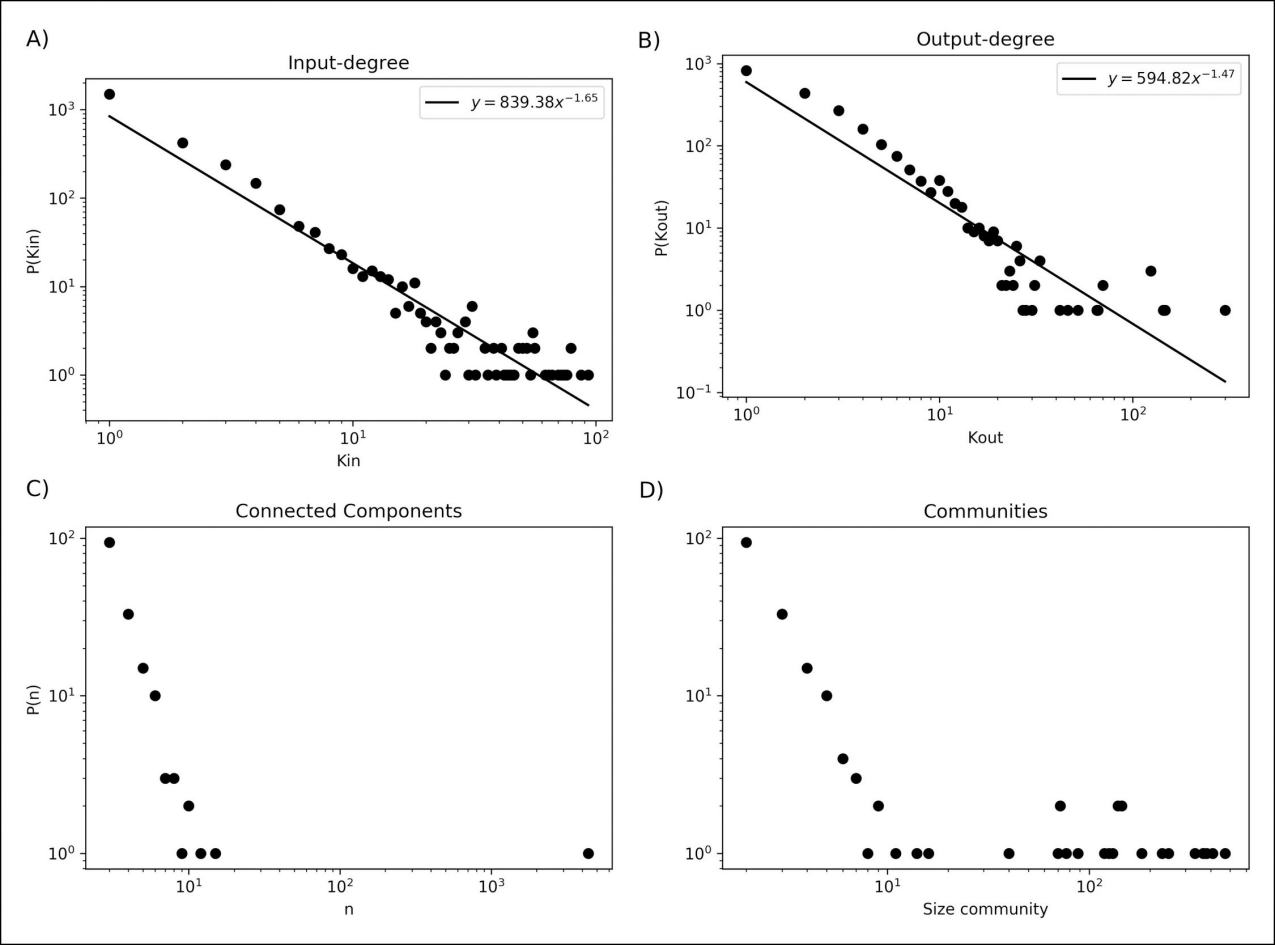
Topological estimations of the network of drugs and targets. A) Input-degree distribution [P(*K*_in_)]. B) Output-degree distribution [P(*K*_out_)]. C) Distribution of the number of nodes in connected components [P(*n*)], where *n* represents the number of nodes and P(*n*) is the probability of finding a component with a specific size. D) Distribution of size communities in the networks, where P(Community) is the probability of finding a community with a specific size.

In order to determine the number of drugs that affect a target (*K*_in_) and the number of targets affected by a drug (*K*_out_), the network was considered a directed network. Based on this network, it was found that each node is connected on average with 3.8 neighbors. In this regard, this network has a consistent distribution of scale-free of nodes degree, with a *γ* < 2 (γ_in_ = 1.6, γ_out_ = 1.47), suggesting that the highest-degree node influences a large fraction of all nodes in the network (Fig 2A and 2B) [29]. Concerning the degree, the network exhibits a decreasing value for *C(k)*, indicating that small groups or modules of elements are well-connected, whereas when the group increases in size the elements are progressively less connected.

In addition, the connectivity or associations between every pair of nodes showed that the drug-target network is constituted by 164 connected components, where one giant component contains 4376 interactions, which represent 89.99% of all the elements (Fig 2C). The giant component contains targets primarily related to metabolism, cardiovascular diseases, and cancer, among others. In contrast, the highest proportion of related components (94) have a size of two elements, *i*. *e*. they only relate a drug to its target. The isolated connected components contain targets related to diverse organisms, such as the bacteria *Escherichia coli*, *Pseudomonas aeruginosa*, and *Mycobacterium tuberculosis*, and human herpesvirus, among other pathogens.

Moreover, degree, closeness, betweenness, and eigenvector centrality were calculated as centrality measures to identify the most important nodes in the drug-target network (Table 1). These metrics are based on the connectivity of the nodes and the shortest paths between each pair of nodes. In this regard, the histamine H1 receptor was found to be the most important node in the centrality of input-degrees. This receptor belongs to the family of rhodopsin-like GPCRs, and it is activated by the biogenic amine histamine. The H1 receptor is linked to an intracellular G-protein (Gq) that activates phospholipase C and the inositol triphosphate (IP3) signaling pathway [39]. In general, these receptors are widely distributed in the brain, smooth muscles from airways, cardiovascular system endothelial cells, and lymphocytes [40]. From the network, it was identified that this node is affected by 93 different drugs, including the antipsychotic drugs loxapine, amitriptyline, clozapine, aripiprazole lauroxil, ziprasidone, aripiprazole, and amoxapine, among others; suggesting a low specificity of these drugs to their targets. In this context, these antipsychotic drugs have also been associated with metabolic side effects, including insulin resistance, glucose intolerance, overeating, increased adiposity, metabolic syndrome, and diabetes, as a consequence of the blocking of H1-histamine receptors [41]. In the case of the centrality of output-degrees, fostamatinib was found to be the most important node, as it interacts with 300 different targets, such as arachidonate 5-lipoxygenase, epidermal growth factor receptor 2, and vascular endothelial growth factor receptor 2. Fostamatinib is a small-molecule spleen tyrosine kinase (Syk) inhibitor for the treatment of rheumatoid arthritis, autoimmune thrombocytopenia, autoimmune hemolytic anemia, IgA nephropathy, and lymphoma [42,43]. Therefore, the large diversity of targets could be because Syk has roles in cellular proliferation, differentiation, survival, immune regulation, and cytoskeletal rearrangements during phagocytosis [43,44].

**Table 1:** The top 10 most influential nodes according to their centralities.

Level	Input-Degree centrality	Output-Degree centrality	Closeness centrality	Betweenness centrality	Eigenvector centrality
1	Histamine H1 receptor (93)	Fostamatinib (300)	Histamine H1 receptor (0.019)	Copper (6.4e-06)	5-Hydroxytryptamine receptor 2A (0.2)
2	Muscarinic acetylcholine receptor M1 (87)	Copper (147)	Muscarinic acetylcholine receptor M1 (0.018)	Prothrombin (3.8e-06)	Alpha-1A adrenergic receptor (0.19)
3	DNA (79)	NADH (144)	DNA (0.016)	Iron (2.2e-06)	D(2) dopamine receptor (0.19)
4	Alpha-1A adrenergic receptor (79)	Zinc (124)	Alpha-1A adrenergic receptor (0.016)	Glutathione (1.8e-06)	Muscarinic acetylcholine receptor M1 (0.17)
5	D(2) dopamine receptor (76)	Zinc acetate (124)	D(2) dopamine receptor (0.016)	Lactose (3.8e-07)	Histamine H1 receptor (0.17)
6	Muscarinic acetylcholine receptor M2 (74)	Zinc chloride (124)	Muscarinic acetylcholine receptor M2 (0.015)	Somatostatin (2.1e-07)	5-Hydroxytryptamine receptor 2C (0.17)
7	5-Hydroxytryptamine receptor 2A (72)	Glutamic Acid (70)	5-Hydroxytryptamine receptor 2A (0.015)	Dabigatran etexilate (2.1e-07)	Loxapine (0.16)
8	Muscarinic acetylcholine receptor M3 (70)	Flavin adenine dinucleotide (70)	Muscarinic acetylcholine receptor M3 (0.014)	Adenosine (1.7e-07)	5-Hydroxytryptamine receptor 1A (0.16)
9	Prostaglandin G/H synthase 2 (66)	Pyridoxal phosphate (66)	Prostaglandin G/H synthase 2 (0.013)	Cystine (1.7e-07)	Alpha-1B adrenergic receptor (0.15)
10	Estrogen receptor alpha (64)	Citric acid (65)	Estrogen receptor alpha (0.013)	Tyrosine (1.7e-07)	Alpha-2A adrenergic receptor (0.15)

*The numbers in parentheses correspond to the value defined by each metric. The largest value corresponds to the most central node.

Furthermore, the *closeness* centrality of node *v*, defined as the reciprocal of the sum of the length of the shortest paths between the node *v* and all other nodes in the graph, *i*.*e*., nodes that are able to spread the information very efficiently through the network, identified the histamine H1 receptor with a centrality value of 0.019, it is the node that minimizes the sum of distances to the other nodes. In contrast, copper with a centrality value of 6.4e-06, was identified as the most significant in terms of the *betweenness* centrality of node *v*, defined as the sum of the fraction of all-pairs shortest paths that pass-through *v*, *i*.*e*., the influence of a vertex over the flow of information between every pair of vertices under the assumption that information primarily flows over the shortest paths between them. Copper is a trace element that is important for the proper functioning of diverse enzymes, including cytochrome *c* oxidase, monoamine oxidase, and superoxide dismutase [45], and as such it is essential to human health. Finally, the 5-hydroxytryptamine receptor 2A, which belongs to the serotonin receptor family and is a GPCR [46], is a receptor for various drugs and psychoactive substances, including mescaline, psilocybin, 1-(2,5-dimethoxy-4-iodophenyl)-2-aminopropane (DOI), and lysergic acid diethylamide (LSD) [47], was the most important node when eigenvector centrality was calculated, with a centrality value of 0.2, where nodes that have a high eigenvector centrality value are connected to many nodes that in turn are well-connected (Table 1).

### Ten hubs define the main structure of the network

In order to identify the most connected nodes associated with the drug-targets network, hubs were determined. To this end, a hub was defined as a drug with connections with many other nodes, *i*.*e*., a large output-degree. In follow, we describe the top of 10 hubs that interact with 33% of the target genes.

**Fig 3:**
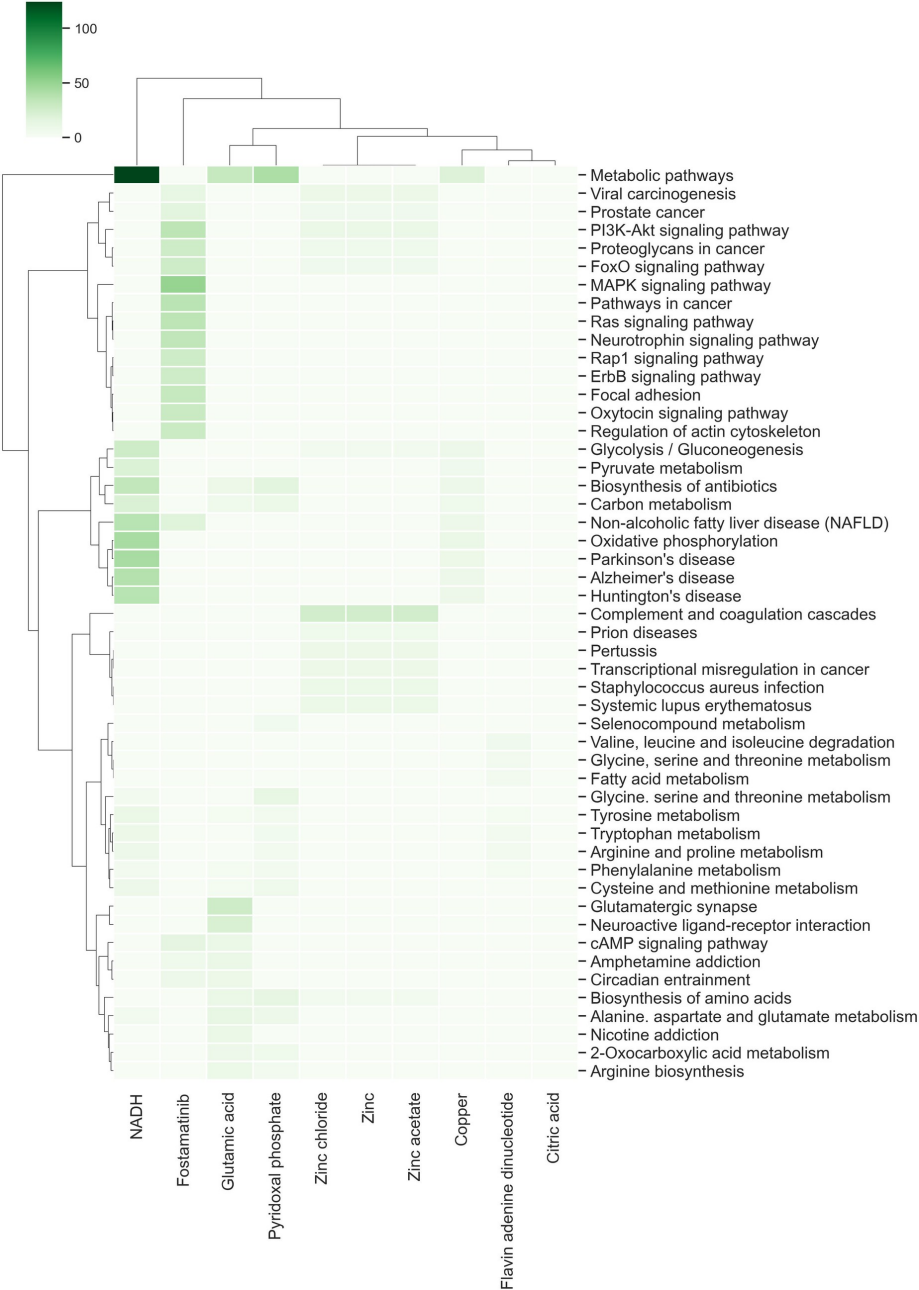
The top metabolic pathways related to the targets affected by hub drugs. The 10 richest metabolic pathways were selected for each hub and were hierarchically clustered based on Euclidean distance and Ward’s method for linkage analysis. Each row represents the KEGG pathways and each column represents hub drugs.

Fostamatinib, which is used for the treatment of chronic immune thrombocytopenia in adults [48], and interacts with 300 targets, was found as the most connected drug. We identified that their targets are related mainly to 10 classes of enriched diseases, among which are metabolic diseases, cancer, chemidependency, and pharmacogenomic, neurological, renal, immune, infectious, and psychiatric diseases. Likewise, we identified that these targets are related to 101 metabolic pathways: MAPK, neurotrophin, ErbB, GnRH and Ras signaling pathways, axon guidance, and focal adhesion, among others (Fig 3 and S2 Table).

Copper identified as a hub, is an essential cofactor for multiplex B redox enzymes involved in respiratory oxidation in both humans and animals [49,50], and it interacts with 147 different target genes mainly associated with cancer and neurological, immune, infectious, and renal diseases, among others.

The other drug also identified as a hub is the reduced nicotinamide adenine dinucleotide (NADH), that is is a small molecule and cofactor of cellular metabolism with a central role in cellular metabolism and energy production as hydride-accepting and hydride-donating coenzymes [51,52] (Fig 3 and S2 Table). NADH interacts with 144 target genes associated with functional classes as: infection, neurological, pharmacogenomics, cancer, aging-related, and psychiatric diseases; probably because NADH impacts different metabolic pathways, such as oxidative phosphorylation, Parkinson’s and Alzheimer’s diseases, among others (Fig 3 and S2 Table).

Zinc, zinc chloride, and zinc acetate are drugs identified in this set of hubs. Zinc is an essential trace element for the appropriate functioning of diverse enzymes and plays an important role in protein synthesis and in cell division. These drugs interact with 124 targets associated with metabolism, cardiovascular, cancer, neurological, and immune responses. It impacts different metabolic pathways, such as the complement and coagulation cascades, infections by the pathogen bacterium *Staphylococcus aureus*, and systemic lupus erythematosus, among others (Fig 3 and S2 Table). Recently, this element has been associated with the pathophysiology and treatment of affective disorders [53].

In addition, Glutamic acid, another drug identified in this set, has been described as the most common excitatory neurotransmitter in the central nervous system. It is conjectured that glutamate is involved in cognitive functions like learning and memory, though excessive amounts may cause neuronal damage associated with diseases like amyotrophic lateral sclerosis, lathyrism, and Alzheimer’s disease. Glutamic acid interacts with 70 targets associated with diverse diseases, such as chemical dependency and neurological infection. Its role impacts different metabolic pathways, including metabolic pathways, glutamatergic synapse, neuroactive ligand-receptor interaction, alanine, aspartate, and glutamate metabolism, and nicotine addiction (Fig 3 and S2 Table).

Another drug found as a hub is Flavin adenine dinucleotide (FAD). This drug is the substituent at position 10 of the flavin nucleus 5’-adenosyldiphosphoribityl group. FAD is approved for use in Japan under the trade name Adeflavin as an ophthalmic treatment for vitamin B2 deficiency diseases such as keratitis and blepharitis [54]. FAD interacts with 70 targets and is associated with diverse diseases, such as cancer, neurological diseases, and chemical dependency. Its role impacts different metabolic pathways including valine, leucine, and isoleucine degradation; glycine, serine, and threonine and fatty acid metabolisms (Fig 3 and S2 Table).

Pyridoxal phosphates (PLP) is the active biochemical form of pyridoxine. It is a coenzyme of amino acid metabolism, particularly for tryptophan and methionine. PLP can be used as a dietary supplement in cases of vitamin B6 deficiency and it is a naturally occurring substance evaluated as a cryoprotectant in myocardial infection, ischemia, and strokes [55]. PLP interacts with 66 targets that are mainly associated with infections and neurological, psychiatric, and developmental diseases, among others. In this regard, the main enriched metabolic pathways identified in the target dataset are Biosynthesis of antibiotics, amino acids, and Glycine, serine, and threonine, and Carbon metabolism (Fig 3 and S2 Table).

Finally, the Citric acid that is formed in the tricarboxylic acid cycle or may be introduced with diet, and it is a key intermediate in metabolism, was also found in this set of hubs. The salts of citric acid (citrates) can be used as anticoagulants due to their calcium-chelating ability [56]. It interacts with 65 targets; however, we did not identify enriched disease classes and metabolic pathways associated with those targets.

### Identification of communities in the network

In order to identify the most highly related elements, we analyzed the network in terms of communities. In this context, a community was defined as a subset of nodes densely connected in comparison with the rest of the network, and as such its identification may help to uncover a priori relations not previously identified [57]. To this end, we used the multilevel algorithm described by Blondel et al. in 2008 [23], that outperforms in comparison with other algorithms as Label propagation [58], Walktrap [59], Spinglass [60], and Edge betweenness algorithms [21], on the set of benchmarks, by taking both accuracy and computing time into account, although the modularity-based methods are known to suffer from the resolution limit of modularity, as it has been recently identified [30]. To estimate the accuracy of this method, Newman and Girvan (2004) introduced a quantitative measure for the quality of network division, called modularity (represented by the Q function) [61]. Thus, a Q = 0 suggests that the number of within-community edges is random, whereas if values close to 1, which is the maximum, indicate networks with strong community structure.

**Fig 4:**
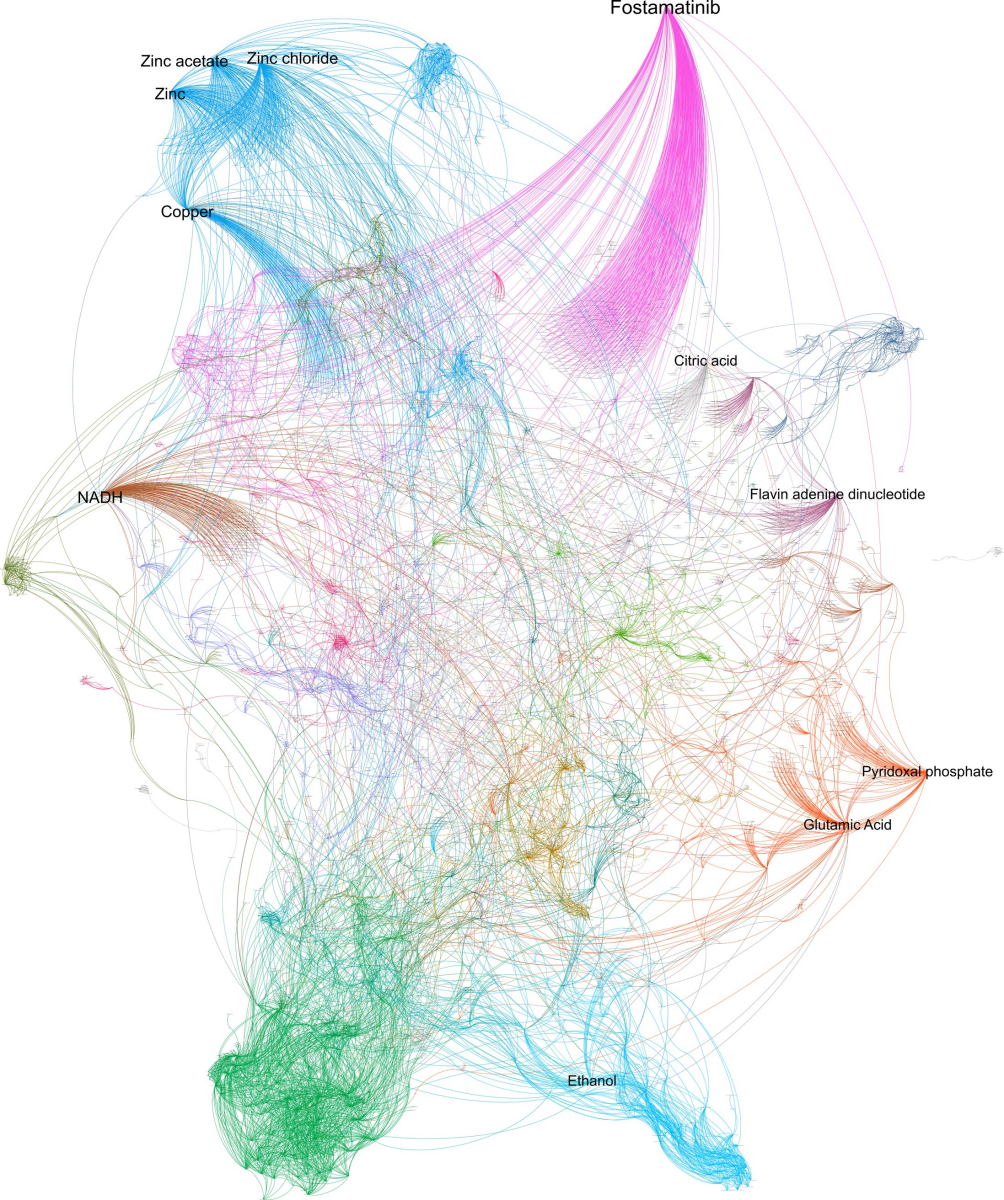
Communities in the drugs-targets network. A network of 4862 nodes and 9286 edges were obtained from the DrugBank database that was clustering in communities. Each community is represented in a different color, and the labels are proportional to the output degree.

Based on this algorithm, we found that the drug-target network contains 187 communities, with modularity of Q = 0.823 (Fig 4), where around 8% of the communities have more than 100 nodes and the largest one ha 471 nodes, whereas 50% are small communities with 2 elements (Fig 2D). From this, in Table 2, the top 10 largest communities are shown; they are mainly related to type 2 diabetes, edema, and rosiglitazone; schizophrenia; Alzheimer’s disease; chronic renal failure; and cancer (S3 Table).

**Table 2:** The top 10 largest communities according to their number of elements.

Community ID*	Number of nodes in the community	Most enrichment disease class **	Number of related enrichment diseases**
3	471	Metabolic and psych	363
1	409	Metabolic and cancer	90
31	382	Pharmacogenomic and cancer	661
34	368	Metabolic and cancer	329
45	367	Metabolic and chemdependency	105
13	335	Metabolic and pharmacogenomic	136
48	230	Infection and neurological	76
66	182	Metabolic and cardiovascular	7
29	145	Cardiovascular and metabolic	19
39	145	Metabolic and cardiovascular	14

* ID is automatically generated for each community.

** Diseases and disease classes with the highest number of related objectives and P-value < 0.05.

### Identification of drug coregulation

In order to identify those drugs that are coregulated in the network, we defined two drugs as being coregulated when they are adjacent to each other and linked to the same target. In this context, the top 10 drugs that share the same target are prescribed for psychiatric diseases such as depression, bipolar disorder, schizophrenia, and psychological depression (Table 3 and S4 Table). An example of such drugs is amoxapine, which coregulates diverse targets with 382 other drugs. This is because their targets are mainly receptor proteins such as the 5-hydroxytryptamine receptor, histamine H1, and H4 receptor, dopamine receptor, alpha-adrenergic receptors, and muscarinic acetylcholine receptors. Although amoxapine is a tetracyclic antidepressant used in patients with neurotic or reactive depressive disorders as well as endogenous and psychotic depressions; the other drugs with which it shares targets are related to alcohol dependence, treatment of allergies and hay fever, viral infection, prostatic hyperplasia, allergic conjunctivitis, and schizophrenia, among others. This finding suggests cross-recognition between amoxapine and the other compounds, as a consequence of the receptor they are recognizing; *i*.*e*. histamine H1 and H2, and dopamine D1 and D2. In this regard, it has been described that these receptors are co-localized and co-mediate the histamine-induced excitation on the two types of neurons [62]. In addition, these receptors are also recognized by other addictive substances, like alcohol or cannabinoids [63]. Therefore, based on the identification of common targets between these drugs, new therapies can be proposed using the existing drugs, as well as, the combination therapies that use pairwise or multiple drugs, being more efficient than monotherapy [64]. Additionally, combination therapy suggests that making side effects less likely, because the doses are lower than in monotherapy therapies [65]. In this context, drug combinations have boosted clinical outcomes for many notoriously complex diseases, such as hypertension, cancer and viral infection, via synergistically targeting multiple disease proteins or pathways [66].

**Table 3:** Top 10 drugs that most co-regulated.

Drug	Treatment*	Number of coregulated drugs**	Most enrichment disease class***
Amoxapine	Major depressive disorder	382	Psych, metabolic, neurological and chemidependency
loxapine	Schizophrenia	363	Metabolic, psych, neurological, pharmacogenomic, and chemidependency
Lamotrigine	Epilepsy and bipolar disorder	339	Psych, neurological, metabolic, pharmacogenomic, and chemidependency
Imipramine	Depression and certain anxiety disorders	332	Psych, chemidependency, metabolic, pharmacogenomic, and neurological
Desipramine	Depression	322	Psych, pharmacogenomic, metabolic, neurological, chemidependency, and cardiovascular
Doxepin	Depressive disorder, anxiety disorders, chronic hives, and trouble sleeping	317	Psych, metabolic, pharmacogenomic, chemidependency, and neurological
Chlorpromazine	Schizophrenia	313	Chemidependency, metabolic, psych, pharmacogenomic, and neurological
Nortriptyline	Depression	308	Psych, pharmacogenomic, chemidependency, metabolic, and neurological
Clozapine	Schizophrenia and Parkinson’s disease	307	Metabolic, psych, neurological, pharmacogenomic, and chemidependency
Amitriptyline	Depressive disorder	301	Psych, metabolic, pharmacogenomic, neurological, and chemidependency

* The main treatment for which the drug is prescribed.

** Number of drugs with which one drug shares one or more targets.

*** Diseases class with the highest number of related objectives and P-value < 0.05.

In the same context, loxapine, which is coregulated with 363 other drugs, is prescribed for manifestations of psychotic disorders such as schizophrenia; whereas the other coregulated drugs include bepotastine (administered for the treatment of itchy eyes), ergotamine (for migraine disorders), and lofexidine (for opioid withdrawal). Mainly, these drugs interact with various receptors, such as the muscarinic acetylcholine receptor, hydroxytryptamine receptor, and sodium-dependent dopamine transporters. This result opens the possibility to explore similarities at the receptor recognition by drugs with not similar effects. In this regard, the dopamine, histamine, and 5-hydroxytryptamine receptors, recognized by diverse drugs already described, share similarity at the structural level, belonging to the superfamily of G-protein-coupled receptor, rhodopsin-like [67].

Meanwhile, lamotrigine is coregulated with 339 other drugs. It is used in the treatment of both epilepsy and as a mood stabilizer in bipolar disorder; whereas the other coregulated drugs include tetrabenazine (prescribed for chorea associated with Huntington’s disease), celiprolol (medication in the class of beta-blockers, used in the treatment for the management of mild to moderate blood pressure), and dextropropoxyphene (for relief of mild to moderate pain), among others. These drugs interact with diverse targets, such as the GABA-A receptor, kappa-type opioid receptor, histamine H1 receptor, and voltage-dependent R-type calcium channel subunit alpha-1E; with no obvious similarity. We consider that these relations could be associated with new interactions to be further evaluated.

In summary, it is necessary to consider the consequences of two drugs having the same target protein, such as xylometazoline, prescribed for nasal congestion, and loxapine, a drug used to treat schizophrenia. In this regard, xylometazoline is an alpha-2A adrenergic receptor agonist, whereas loxapine is an alpha-2A adrenergic receptor binder, both receptor proteins belong to the superfamily of G-protein-coupled receptor, rhodopsin-like (IPR000276) [67,68], we hypothesize that receptor similarity is influencing the cross-recognition of diverse drugs that must be extensively explored.

To determine a minimum dominant set of drugs that can affect the network, we selected the giant component with 1930 drugs, 2445 targets, and 8948 edges. Subsequently, we carried out an iterative random exclusion of one drug at a time for 100,000 times, if the eliminated node did not decrease the interaction with all the targets, it was eliminated from the dominant set and if this decreased the prediction of the targets, the node was preserved. From this analysis, we identified a subset of 433 out of 1930 drugs that is susceptible to affecting the giant component of the network, representing a good opportunity for further exploration of new interactions. Hereof, new interactions could be identified on the basis for network-based drug repositioning, which considers the proteins that are localized in the corresponding disease module o sub-network within the human Protein–protein interactions network [69], and proteins that serve as drug targets for a specific disease may also be suitable for another disease and drugs [70]. This approach has the advantage of mitigating the costs and risks associated with the early development stage, and shortening routes to approval for therapeutic indication [71–73].

## Conclusions

Recent advances in the understanding of the interactions of drugs and targets provide a framework to explore probable new interactions previously not detected. In this work, we identified an interesting set of drugs in the network. The first one corresponds to the drugs that interact with the most targets, where the most connected is fostamatinib, a drug used for the treatment of chronic immune thrombocytopenia in adults. Indeed, this compound affects a large number of targets, inhibiting signal transduction by Fcγ receptors involved in the antibody-mediated destruction of platelets in chronic ITP or in the inhibition of T- and B-lymphocyte activation by T-cell receptors and B-cell receptors, respectively [74,75].

Additionally, nine identified hubs (drugs) acting as cofactors or coenzymes, are related to dietary and nutritional therapies: copper, NADH, zinc, zinc acetate, zinc chloride, glutamic acid, FAD, pyridoxal 5’ phosphate, and citric acid. Their high connectivity may be due to their importance at the metabolic level; in the case of copper, it is incorporated into many oxidase enzymes as a cofactor; or Zinc, that is associated with hundreds of proteins that transport and traffic this element. Recently, Zinc exhibits antiviral activity against a variety of viruses, such as herpes simplex virus and the common cold [76], opening the opportunity to evaluate this element against viral diseases.

Glutamic acid is the most widespread neurotransmitter in brain function, as an excitatory neurotransmitter, and as a precursor for the synthesis of GABA in GABAergic neurons. Pyridoxal 5’-phosphate (PLP) is necessary for the enzymatic reaction governing the release of glucose from glycogen and acts as a coenzyme in all transamination reactions and in some oxidation and deamination reactions of amino acids. Finally, citric acid is a weak acid that is formed in the tricarboxylic acid cycle, used as an anticoagulant, it is one of the active ingredients in Phexxi, a non-hormonal contraceptive agent that was approved by the FDA.

In the same sense, we identified highly connected sets of nodes that were structured in communities; the largest communities have more than 400 elements and are related to metabolic diseases, such as psychiatric disorders and cancer. In this regard, drugs used for schizophrenia treatment exhibit a wide diversity of targets, suggesting that diverse side effects could emerge.

**Fig 5:**
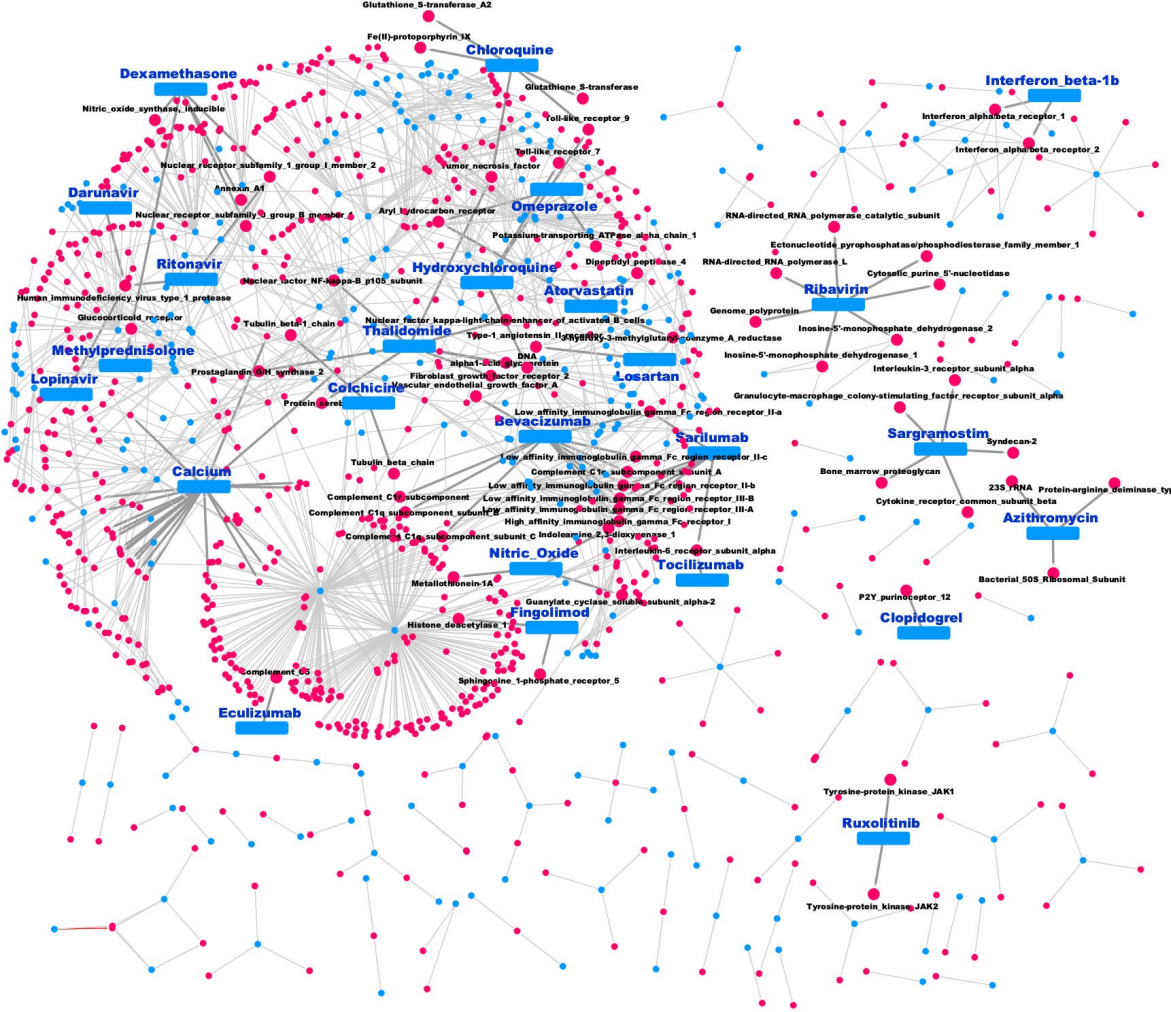
Viral drugs-targets network. A total of 1140 nodes and 1386 edges were related to viral diseases. Targets are indicated as blue nodes, and drugs are the red nodes; Drugs related to Coronavirus infections are indicated as yellow nodes.

The FDA has only approved 12 new antivirals from 2012 to 2017, thus network-based strategies can help this lack of drugs. In this context, a group of 316 drugs of interest are related to virus infections; they are related to 824 targets (Fig 5). Based on this approach, it was identified that the main drugs are grouped in the communities 0 and 3, which share the target with other related drugs to type 2 diabetes, edema; prostate cancer; chronic renal failure; breast cancer, and colorectal cancer.

Finally, we identified a subset of 25 drugs which have been reported for the tentative treatment of SARS-CoV-2 (https://clinicaltrials.gov/), which includes atorvastatin, chloroquine, darunavir, hydroxychloroquine, interferon beta-1b, lopinavir, dexamethasone, and tocilizumab, among others (Fig 5). In particular, dexamethasone that reduces deaths caused by SARS-CoV-2 [77], interacts with diverse targets, associated to steroid or nuclear hormone receptors as Glucocorticoid receptor or Nuclear receptor subfamily 0 group B member 1 and Nuclear receptor subfamily 1 group I member 2, and Annexin A, and, Nitric oxide synthase, involved in catalyzing the production of NO from L-arginine. NO is an important cellular signaling molecule [78]. Thus, our approach opens the possibility to explore these compounds and their targets to contend against viral diseases, such as those re-emergent viruses, such as Zika, Ebola, and Middle East respiratory syndrome coronavirus that are characterized by pandemic potential [79]. Thus, different scenarios of antiviral drug repurposing have been previously suggested, such as the: same target—new virus; i. e. when an antiviral drug with a specific viral or cellular function/pathway target is found to possess activity against other viruses; such as favipiravir used against influenza virus showed repurposing potential against Ebola and Zika. A second case involves, same target—new indication, that occurs when a pharmacological target is found to be essential in a pathogenic process associated with a viral infection, and the approved drug can be also exploited as an antiviral therapeutic agent (new indication); such as occurs with the anticancer drug imatinib that also acts against pathogenic coronaviruses [80]. Finally, a new target—new indication, which occurs when an approved drug with established bioactivity in a specific pathway or mechanism is found to have a new molecular target which is essential for virus replication; such as the antimicrobial agents (e.g., teicoplanin, and ivermectin, and nitazoxanide) that also inhibit the viral replication in infected cells [81,82].

We consider that the approach described in this work could be also extended to the identification of possible microRNAs targets, such as it has been previously proposed for Alzheimer’s disease [83] and prostate cancer [84].

## Supporting information

S1 Table Collection of drug-targets interactions. (XLSX)

S2 Table Top 10 hub drugs and enrichment analysis. (XLSX)

S3 Table Collection of communities in the network and enrichment analysis. (XLSX)

S4 Table Collection of drug co-regulation and enrichment analysis. (XLSX)

S1 File Scripts of python and octave. (PDF)
