# Supplementary material for: A landscape for drug-target interactions based on network analysis
Source: PLoS One. 2021 Mar 17;16(3):e0247018. doi: 10.1371/journal.pone.0247018 (PMC7968663; doi:10.1371/journal.pone.0247018)
Supplement: S1 File — (PDF) [file pone.0247018.s005.pdf]

A landscape for drug-target interactions based on network analysis

Programs used:

```
%%%%%%%%%%%%%%%%%%%%%%%%%%%%%%%%%%%%%%%%%%%%%%%%%%%%%%%%%%%%%%%%%%
%%
%% Program that calculates the co-regulations of a directed
network from an adjacency matrix
%%%%%%%%%%%%%%%%%%%%%%%%%%%%%%%%%%%%%%%%%%%%%%%%%%%%%%%%%%%%%%%%%%
%%

t0 = clock ();
%%%Input%%%%%%%%%%%%%%%%%%%%%%%%%%%%%%%%%%%%%%%%%%%%%%%%%%%%%%%%%

%Matrix
load 'Matriz3';

%Directed matrix without loops
Matriz3B=Matriz3;
for gy=1:1:rows(Matriz3B)
Matriz3B(gy,gy)=0;
endfor

%%%undirected matrix
MatrizN=sign(Matriz3B+Matriz3B');


%%%%Variables
vv=[];

%%%% feature:
%%Include feature lists, these cases are all 1.
load 'listacar.txt';
v=listacar;

din=[];
dout=[];
deg=[];
%%%%grados de entrada y salida
din=sum(sign(Matriz3B));
dout=sum(sign(Matriz3B'));
deg=sum(MatrizN);

for i=1:1:columns(dout)

    if dout(1,i)~=0
    a1=rows(find(v(find(Matriz3B(i,:)==1))==1));
```

```
    a2=rows(find(v(find(Matriz3B(i,:)==1))==2));
    a3=rows(find(v(find(Matriz3B(i,:)==1))==3));
    a4=rows(find(v(find(Matriz3B(i,:)==1))==4));
    a5=rows(find(v(find(Matriz3B(i,:)==1))==5));
    vv=[vv;i,dout(1,i),a1,a2,a3,a4,a5];
    end
endfor


%%% 3. coregulation

listatemp=zeros(1,max(vv(:,2))+1);
v2=[];
Mfrecc=zeros(rows(Matriz3B),columns(Matriz3B));
Mtipo=zeros(rows(Matriz3B),columns(Matriz3B));
lista2=[];

for j=1:1:rows(vv)
          r=[find(Matriz3B(vv(j,1),:)~=0)];
          listatemp=[listatemp;vv(j,1),r,zeros(1,max(vv(:,2))-
columns(r))];
endfor


    for k=2:1:rows(listatemp)-1
    for l=2:1:columns(listatemp)
    if (listatemp(k,l)~=0)
     for m=k+1:1:rows(listatemp)


    if(columns(find(listatemp(m,2:columns(listatemp))==listatemp(k
,l)))~=0)

           v2=[v2;listatemp(k,1),listatemp(m,1),listatemp(k,l)];
           save nombre v2
           rename('nombre',['listacorreg']);
     end
     endfor
    end
    endfor
    endfor


for n=1:1:rows(v2)
Mfrecc(v2(n,1),v2(n,2))=Mfrecc(v2(n,1),v2(n,2))+1;
Mtipo(v2(n,1),v2(n,2))=v(v2(n,2),1);
Mtipo(v2(n,2),v2(n,1))=v(v2(n,1),1);
endfor
```

```
for x=1:1:rows(Mfrecc)
for y=1:1:columns(Mfrecc)
if Mfrecc(x,y)~=0
lista2=[lista2;x,y,Mfrecc(x,y),v(x,1),v(y,1)];
end
endfor
endfor
    save nombre lista2
          rename('nombre',['listacorreg2']);




printf("%f segundos transcurridos\n",etime (clock (), t0))


%%%%%%% OUTPUT %%%%%%%%%%%%%%%%%%%%%%%%%%%%%%%%%%
%'listacorreg' (Durg 1          Drug 2          who regulates?)
%'listacorreg2' (Durg 1    Durg 2          How often do they co-
regulate?)
%%%%%%%%%%%%%%%%%%%%%%%%%%%%%%%%%%%%%%%%%%%%%%%%
```

%%%
%%
%% **Program that searches for a set of dominants given an adjacency matrix of a directed network**
%%%
%%

```
%Read an Adjacency matrix
load Matriz3.txt
Nv=rows(Matriz3);
A=Matriz3;
G=A;

A=abs(sign(G));
% % input degree
Din=sum(abs(A));
%Grados de salida
B=A';
Dout=sum(abs(B));

Vdom=[];
```

```
Lv=ones(1,Nv);
Vd=ones(1,Nv);


Drug=find(Dout>0);

%Find nodes with output degree zero
coorcero=find(Din==0);
Lv(coorcero)=0;


totalnodes=columns(Lv);
totaltargets=columns(find(Lv==1));
Diff=totalnodes-totaltargets;
porcen=0;

%Identifies a set of nodes that generates all targets

while porcen !=100
  ant=porcen;
  elegido=Drug(randi(size(Drug,2),1));
  Vdom=[Vdom,elegido];
  for g=Vdom
    generados=find(A(g,:)~=0 );
    Lv(generados)=0;
    Identif=columns(find(Lv==1));
    Identif=columns(find(Lv==1));
    porcen= (1-(Identif/totaltargets))*100;
  end
  if porcen <= ant
    Vdom(end)=[];
  end
end


%Searches for a minimum set of drugs that affect the total targets.

for i=1:1:100000
  NVdom=Vdom;
  Lv=ones(1,Nv);
  coorcero=find(Din==0);
  Lv(coorcero)=0;
  elegido=NVdom(randi(size(NVdom,2),1));
  NVdom(NVdom==elegido)=[];
  for g=NVdom
    generados=find(A(g,:)~=0 );
    Lv(generados)=0;
    Identif=columns(find(Lv==1));
```

```
    porcen= (1-(Identif/totaltargets))*100;
  end

  if porcen == 100
    Vdom=NVdom;
  end
end
Z1=Vdom;

save nombre Vdom
rename('nombre',['Vdom100.txt']);


%%%%%%% OUTPUT %%%%%%%%%%%%%%%%%%%%%%%%%%%%%%%%%%
%'Vdom100' (Set of nodes that interacted with all targets)
%%%%%%%%%%%%%%%%%%%%%%%%%%%%%%%%%%%%%%%%%%%%%%%%
```

Topology of Drugs-Targets network

A landscape for drug-target interactions based on network analysis

In [1]:

```
import os
import pandas as pd
import numpy as np
import networkx as nx
from networkx.algorithms import bipartite
import matplotlib.pyplot as plt
import scipy as sp
print (os.getcwd())
os.chdir("/Users/galanve/Desktop/PosdocIIMAS/Proyectos/Farmacos/")
```

```
/Users/galanve/Desktop/PosdocIIMAS/Proyectos/Farmacos
```

Read the list of interactions

In [2]:

```
Drug1=pd.read_csv('Drug-targetv1.txt', sep='\t', header=None,names=['Drug','Edg
e','Targer1','Details'])
Drug1.head()
```

Out[2]:

	Drug	Edge	Targer1	Details
0	Lepirudin	target	Prothrombin	Details
1	Cetuximab	target	Epidermal_growth_factor_receptor	Details
2	Cetuximab	target	Complement_C1r_subcomponent	Details
3	Cetuximab	target	Low_affinity_immunoglobulin_gamma_Fc_region_re...	Details
4	Cetuximab	target	Low_affinity_immunoglobulin_gamma_Fc_region_re...	Details

Build the network from the list of interactions

In [3]:

```
G = nx.from_pandas_edgelist(Drug1, 'Drug', 'Targer1', create_using=nx.DiGraph)
```

Information of network

In [4]:

```
N,K=G.order(), G.size()
avg_deg=float(K)/N
```

In [5]:

```
print('Nodes:',N)
```

```
Nodes: 4862
```

In [6]:

```
print('Edges:',K)
```

```
Edges: 9286
```

Transform of digraph to grap

In [7]:

```
G2 = G.to_undirected()
```

In [8]:

```
# If your Graph has more than one component, this will return False:
print(nx.is_connected(G2))
```

```
False
```

In [9]:

```
components = nx.connected_components(G2)
largest_component = max(components, key=len)
NumCC=nx.number_connected_components(G2)
```

In [10]:

```
print("Number of Connected components:", NumCC)
```

```
Number of Connected components: 164
```

In [11]:

```
largest_cc = max(nx.connected_components(G2), key=len)
```

In [12]:

```
print('Giant component:',len(largest_cc)+1)
```

```
Giant component: 4376
```

In [13]:

```
# Create a "subgraph" of just the largest component
# Then calculate the diameter of the subgraph, just like you did with density.
subgraph = G2.subgraph(largest_component)
diameter = nx.diameter(subgraph)
print("Network diameter of largest component:", diameter)
```

```
Network diameter of largest component: 18
```

In [14]:

```
comp=[]
for component in nx.connected_components(G2):
    comp.append(len(component)+1)
```

In [15]:

```
CCvalue = np.bincount(comp)
yCC = np.array(CCvalue)
xCC = np.array(range(len(CCvalue)))
```

In [16]:

```
dfCC = pd.DataFrame({'xCC': xCC, 'yCC': yCC}, columns=['xCC', 'yCC'])
dfCC = dfCC[~(dfCC == 0).any(axis=1)]
dfCC.head()
```

Out[16]:

	xCC	yCC
**3**	3	94
**4**	4	33
**5**	5	15
**6**	6	10
**7**	7	3

In [17]:

```
x=dfCC[['xCC']].to_numpy()
y=dfCC[['yCC']].to_numpy()
dflog=np.log(dfCC[['xCC','yCC']])
new_df = dflog.replace([np.inf, -np.inf], np.nan).dropna(axis=0)
```

In [18]:

```
logx = new_df.xCC
logy = new_df.yCC
coeffs = np.polyfit(logx,logy,deg=1)
poly = np.poly1d(coeffs)
yfit = lambda x: np.exp(poly(np.log(x)))
plt.loglog(x,y,'ko')
plt.ylabel('P(n)')
plt.xlabel('n')
plt.title('Connected Components')
#plt.loglog(x,yfit(x),'k')
plt.savefig('DistCC.png',dpi=300)
plt.show()
```

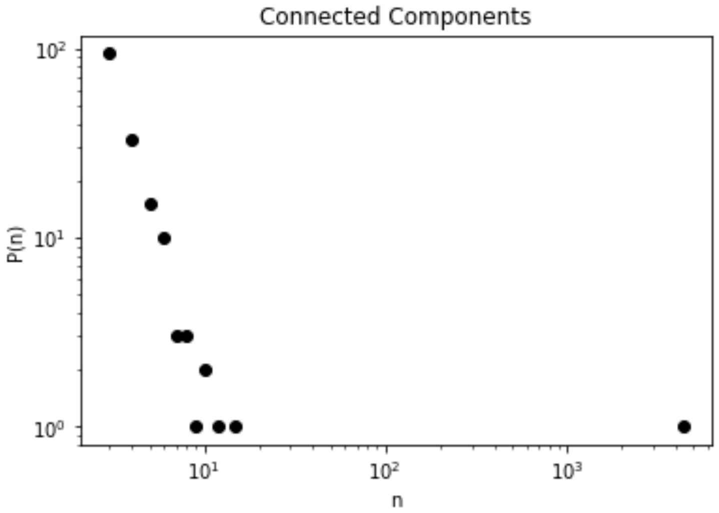

Adjacency matrix

In [19]:

```
M = nx.to_scipy_sparse_matrix(G)
```

Input degree and output degree

In [20]:

```
indegrees = M.sum(0).A[0]
outdegrees = M.sum(1).T.A[0]
```

In [21]:

```
in_degrees = G.in_degree()
out_degrees=G.out_degree()
```

In [22]:

```
Indegree_dict = dict(G.in_degree(G.nodes()))
nx.set_node_attributes(G, Indegree_dict, 'degree')
```

In [23]:

```
import operator
sorted_Indegree = sorted(Indegree_dict.items(), key=operator.itemgetter(1), reve
rse=True)
```

Top 20 nodes by Input degree<\h4>

In [24]:

```
for d in sorted_Indegree[:20]:
    print(d)
```

```
('Histamine_H1_receptor', 93)
('Muscarinic_acetylcholine_receptor_M1', 87)
('DNA', 79)
('Alpha-1A_adrenergic_receptor', 79)
('D(2)_dopamine_receptor', 76)
('Muscarinic_acetylcholine_receptor_M2', 74)
('5-hydroxytryptamine_receptor_2A', 72)
('Muscarinic_acetylcholine_receptor_M3', 70)
('Prostaglandin_G/H_synthase_2', 66)
('Estrogen_receptor_alpha', 64)
('Alpha-2A_adrenergic_receptor', 62)
('Alpha-1B_adrenergic_receptor', 56)
('Sodium-dependent_noradrenaline_transporter', 56)
('5-hydroxytryptamine_receptor_1A', 55)
('Nuclear_receptor_subfamily_1_group_I_member_2', 55)
('Prostaglandin_G/H_synthase_1', 55)
('Glucocorticoid_receptor', 54)
('Beta-2_adrenergic_receptor', 52)
('Sodium-dependent_serotonin_transporter', 52)
('Beta-1_adrenergic_receptor', 50)
```

Top 20 nodes by Output degree<\h4>

In [25]:

```
Outdegree_dict = dict(G.out_degree(G.nodes()))
nx.set_node_attributes(G, Outdegree_dict, 'degree')
sorted_Outdegree = sorted(Outdegree_dict.items(), key=operator.itemgetter(1), re
verse=True)
```

In [26]:

```
for d in sorted_Outdegree[:20]:
    print(d)
```

```
('Fostamatinib', 300)
('Copper', 147)
('NADH', 144)
('Zinc', 124)
('Zinc_acetate', 124)
('Zinc_chloride', 124)
('Glutamic_Acid', 70)
('Flavin_adenine_dinucleotide', 70)
('Pyridoxal_phosphate', 66)
('Citric_acid', 65)
('Ethanol', 52)
('Flavin_mononucleotide', 46)
('Glutathione', 42)
('Loxapine', 33)
('Calcium_Citrate', 33)
('Calcium_Phosphate', 33)
('Calcium_phosphate_dihydrate', 33)
('Zonisamide', 31)
('Glycine', 31)
('Isopropyl_alcohol', 30)
```

Distributions of input and output degree<\h4>

In [27]:

```
indegree_distribution = np.bincount(indegrees)
outdegree_distribution = np.bincount(outdegrees)
```

Distribution of Indput-degree <\h5>

In [28]:

```
xdata = np.array(range(len(indegree_distribution)))
ydata = np.array(indegree_distribution)

dataset = pd.DataFrame({'xdata': xdata, 'ydata': ydata}, columns=['xdata', 'ydat
a'])
dataset = dataset[~(dataset == 0).any(axis=1)]
dataset.head()
```

Out[28]:

	xdata	ydata
**1**	1	1493
**2**	2	422
**3**	3	239
**4**	4	147
**5**	5	74

In [29]:

```
x=dataset[['xdata']].to_numpy(float)
y=dataset[['ydata']].to_numpy(float)
x.astype(float);
y.astype(float);
```

In [30]:

```
dflog=np.log(dataset[['xdata','ydata']])
new_df = dflog.replace([np.inf, -np.inf], np.nan).dropna(axis=0)
```

In [31]:

```
logx = new_df.xdata
logy = new_df.ydata
coeffs = np.polyfit(logx,logy,deg=1)
poly = np.poly1d(coeffs)
yfit = lambda x: np.exp(poly(np.log(x)))
plt.loglog(x,y,'ko')
plt.ylabel('P(Kin)')
plt.xlabel('Kin')
plt.title('Input-degree')
plt.loglog(x,yfit(x),'k', label=r'$y=839.38x^{-1.65}$')
plt.legend()
plt.savefig('InputDegree.png',dpi=300)
plt.show()
```

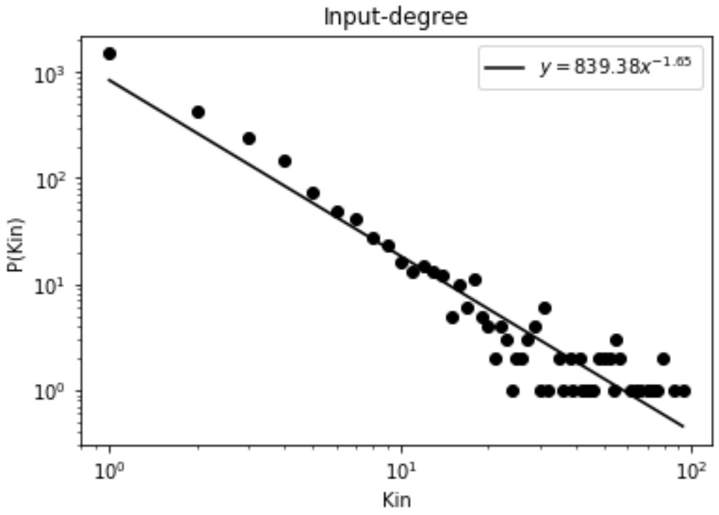

Distribution of Output-degree <\h5>

In [32]:

```
xdata = np.array(range(len(outdegree_distribution)))
ydata = np.array(outdegree_distribution)

dataset = pd.DataFrame({'xdata': xdata, 'ydata': ydata}, columns=['xdata', 'ydat
a'])
dataset = dataset[~(dataset == 0).any(axis=1)]
dataset.head()
```

Out[32]:

	xdata	ydata
**1**	1	822
**2**	2	437
**3**	3	268
**4**	4	160
**5**	5	104

In [33]:

```
x=dataset[['xdata']].to_numpy(float)
y=dataset[['ydata']].to_numpy(float)
x.astype(float);
y.astype(float);
```

In [34]:

```
dflog=np.log(dataset[['xdata','ydata']])
new_df = dflog.replace([np.inf, -np.inf], np.nan).dropna(axis=0)
```

In [35]:

```
logx = new_df.xdata
logy = new_df.ydata
coeffs = np.polyfit(logx,logy,deg=1)
poly = np.poly1d(coeffs)
yfit = lambda x: np.exp(poly(np.log(x)))
plt.loglog(x,y,'ko')
plt.ylabel('P(Kout)')
plt.xlabel('Kout')
plt.title('Output-degree')
plt.loglog(x,yfit(x),'k',label=r'$y=594.82x^{-1.47}$')
plt.legend()
plt.savefig('OutputDegree.png',dpi=300)
plt.show()
```

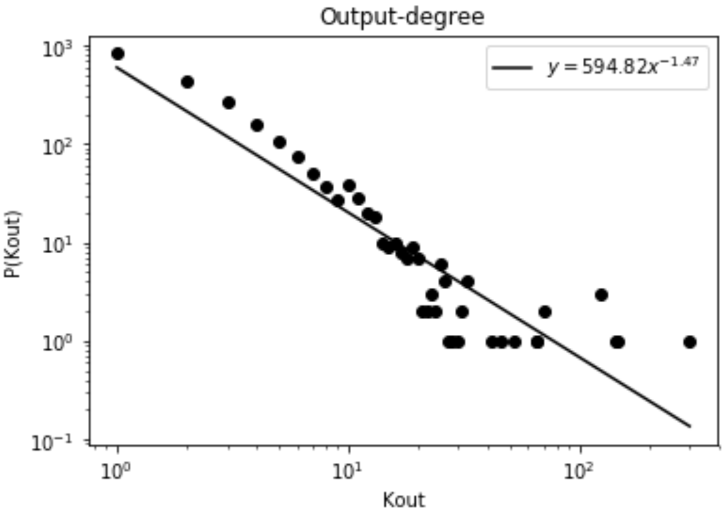

Top 20 nodes by Degree centrality

In [36]:

```
DegreeCen=nx.degree_centrality(G)
sortedDCen = sorted(DegreeCen.items(), key=operator.itemgetter(1), reverse=True)
for d in sortedDCen[:20]:
    print(d)
```

```
('Fostamatinib', 0.061715696358773914)
('Copper', 0.030446410203661796)
('NADH', 0.029623534252211477)
('Zinc', 0.025509154494959885)
('Zinc_acetate', 0.025509154494959885)
('Zinc_chloride', 0.025509154494959885)
('Histamine_H1_receptor', 0.019131865871219914)
('Muscarinic_acetylcholine_receptor_M1', 0.017897551944044434)
('DNA', 0.016251800041143797)
('Alpha-1A_adrenergic_receptor', 0.016251800041143797)
('D(2)_dopamine_receptor', 0.01563464307755606)
('Muscarinic_acetylcholine_receptor_M2', 0.015223205101830898)
('5-hydroxytryptamine_receptor_2A', 0.014811767126105739)
('Muscarinic_acetylcholine_receptor_M3', 0.01440032915038058)
('Glutamic_Acid', 0.01440032915038058)
('Flavin_adenine_dinucleotide', 0.01440032915038058)
('Prostaglandin_G/H_synthase_2', 0.013577453198930261)
('Pyridoxal_phosphate', 0.013577453198930261)
('Citric_acid', 0.013371734211067682)
('Estrogen_receptor_alpha', 0.013166015223205102)
```

Top 20 node by Betweenness centrality

In [37]:

```
betweenness_dict=nx.betweenness_centrality(G)
sorted_betweenness = sorted(betweenness_dict.items(), key=operator.itemgetter(1
), reverse=True)
for d in sorted_betweenness[:20]:
    print(d)
```

```
('Copper', 6.391680487088382e-06)
('Prothrombin', 3.809610886344069e-06)
('Iron', 2.2011085121099063e-06)
('Glutathione', 1.777818413627232e-06)
('Lactose', 3.809610886344069e-07)
('Somatostatin', 2.1164504924133716e-07)
('Dabigatran_etexilate', 2.1164504924133716e-07)
('Adenosine', 1.6931603939306973e-07)
('Cystine', 1.6931603939306973e-07)
('Tyrosine', 1.6931603939306973e-07)
('Nitric_Oxide', 1.269870295448023e-07)
('Digoxin', 4.232900984826743e-08)
('Methotrexate', 4.232900984826743e-08)
('Lepirudin', 0.0)
('Cetuximab', 0.0)
('Epidermal_growth_factor_receptor', 0.0)
('Complement_C1r_subcomponent', 0.0)
('Low_affinity_immunoglobulin_gamma_Fc_region_receptor_II-c', 0.0)
('Low_affinity_immunoglobulin_gamma_Fc_region_receptor_III-B', 0.0)
('Low_affinity_immunoglobulin_gamma_Fc_region_receptor_III-A', 0.0)
```

Top 20 node by Closeness centrality

In [38]:

```
closeness_dict=nx.closeness_centrality(G)
sorted_closeness = sorted(closeness_dict.items(), key=operator.itemgetter(1), re
verse=True)
for d in sorted_closeness[:20]:
    print(d)
```

```
('Histamine_H1_receptor', 0.019131865871219914)
('Muscarinic_acetylcholine_receptor_M1', 0.017897551944044434)
('DNA', 0.016251800041143797)
('Alpha-1A_adrenergic_receptor', 0.016251800041143797)
('D(2)_dopamine_receptor', 0.01563464307755606)
('Muscarinic_acetylcholine_receptor_M2', 0.0152232051018309)
('5-hydroxytryptamine_receptor_2A', 0.01481176712610574)
('Muscarinic_acetylcholine_receptor_M3', 0.01440032915038058)
('Prostaglandin_G/H_synthase_2', 0.013577453198930261)
('Estrogen_receptor_alpha', 0.013166015223205102)
('Alpha-2A_adrenergic_receptor', 0.012754577247479942)
('Alpha-1B_adrenergic_receptor', 0.011520263320304465)
('Sodium-dependent_noradrenaline_transporter', 0.011520263320304465)
('5-hydroxytryptamine_receptor_1A', 0.011314544332441884)
('Nuclear_receptor_subfamily_1_group_I_member_2', 0.0113145443324418
84)
('Prostaglandin_G/H_synthase_1', 0.011314544332441884)
('Glucocorticoid_receptor', 0.011108825344579305)
('Beta-2_adrenergic_receptor', 0.010697387368854146)
('Sodium-dependent_serotonin_transporter', 0.010697387368854146)
('Beta-1_adrenergic_receptor', 0.010285949393128985)
```

Top 20 node by Eigenvector centrality

In [39]:

```
eigenvector_dict=nx.eigenvector_centrality(G2)
sorted_eigenvector = sorted(eigenvector_dict.items(), key=operator.itemgetter(1
), reverse=True)
for d in sorted_eigenvector[:20]:
    print(d)
```

```
('5-hydroxytryptamine_receptor_2A', 0.20235899295111773)
('Alpha-1A_adrenergic_receptor', 0.19095426071193475)
('D(2)_dopamine_receptor', 0.1868823650743956 8)
('Muscarinic_acetylcholine_receptor_M1', 0.16919254693391497)
('Histamine_H1_receptor', 0.16894664916879285)
('5-hydroxytryptamine_receptor_2C', 0.16709989533183234)
('Loxapine', 0.15695099740290336)
('5-hydroxytryptamine_receptor_1A', 0.15601862694239532)
('Alpha-1B_adrenergic_receptor', 0.1547305544222005)
('Alpha-2A_adrenergic_receptor', 0.1533828617945145)
('Muscarinic_acetylcholine_receptor_M3', 0.1503987743102554)
('Muscarinic_acetylcholine_receptor_M2', 0.14943628234246886)
('Quetiapine', 0.14525900306194722)
('Aripiprazole', 0.14116977680895113)
('Aripiprazole_lauroxil', 0.14116977680895113)
('Ziprasidone', 0.14116977680895113)
('Clozapine', 0.14015592985874709)
('D(1A)_dopamine_receptor', 0.1285353785871056)
('Muscarinic_acetylcholine_receptor_M4', 0.12549685834930102)
('Alpha-2B_adrenergic_receptor', 0.12258451053393431)
```

Communities

In [40]:

```
Comun=pd.read_csv('comunidades.txt', sep='\t', header=None,names=['Community',
'Size'])
Comun.head()
```

Out[40]:

	Community	Size
**0**	0	247
**1**	1	409
**2**	2	4
**3**	3	471
**4**	4	2

In [41]:

```
data=Comun.Size
data1 = np.bincount(data)
```

In [42]:

```
xdata = np.array(range(len(data1)))
ydata = np.array(data1)

dataset = pd.DataFrame({'xdata': xdata, 'ydata': ydata}, columns=['xdata', 'ydat
a'])
dataset = dataset[~(dataset == 0).any(axis=1)]
dataset.head()
```

Out[42]:

	xdata	ydata
**2**	2	94
**3**	3	33
**4**	4	15
**5**	5	10
**6**	6	4

In [43]:

```
x=dataset[['xdata']].to_numpy(float)
y=dataset[['ydata']].to_numpy(float)
x.astype(float);
y.astype(float);
```

In [44]:

```
dflog=np.log(dataset[['xdata','ydata']])
new_df = dflog.replace([np.inf, -np.inf], np.nan).dropna(axis=0)
```

In [45]:

```
logx = new_df.xdata
logy = new_df.ydata
coeffs = np.polyfit(logx,logy,deg=1)
poly = np.poly1d(coeffs)
yfit = lambda x: np.exp(poly(np.log(x)))
plt.loglog(x,y,'ko')
plt.ylabel('P(Community)')
plt.xlabel('Size community')
plt.title('Communities')
#plt.loglog(x,yfit(x),'k')
#plt.loglog(x,yfit(x),'k')
#plt.legend()
plt.savefig('DistrubComm.png',dpi=300)
plt.show()
```

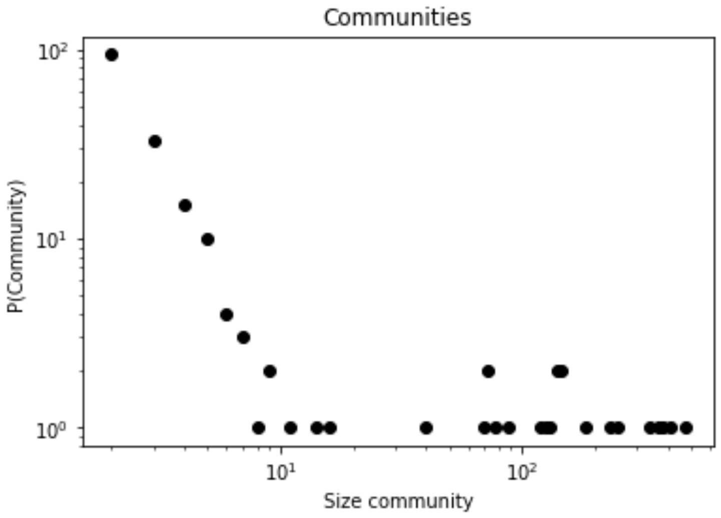

In [ ]:
